# Assessing Autonomic Regulation Under Stress with the Yale Pain Stress Test in Social Drinking and Alcohol Use Disorder

**DOI:** 10.3390/bs15121732

**Published:** 2025-12-15

**Authors:** Shaina Barreto, Colleen McGowan, Nia Fogelman, Rajita Sinha, Stephanie E. Wemm

**Affiliations:** 1 Every Cure, Boston, MA 02142, USA; 2 Department of Psychiatry and Behavioral Sciences, University of Minnesota, Minneapolis, MN 55414, USA; 3 Department of Psychology, Cognitive and Brain Science, University of Minnesota, Minneapolis, MN 55414, USA; 4 Yale Stress Center, Yale School of Medicine, New Haven, CT 06519, USA

**Keywords:** heart rate variability, alcohol use disorder, autonomic function, stress

## Abstract

High levels of stress and individual differences in acute stress responses are important predictors of chronic illness. This study examined the effects of stress and Alcohol Use Disorder (AUD) on heart rate variability (HRV) using the Yale Pain Stress Test (YPST), adapted from the well-established Cold Pressor Test (CPT). The YPST characterized three key (time periods) phases of the cardiovascular stress response to repeated trials of a pain-stress versus no pain-stress control condition: pre-stress baseline, acute reactivity, and recovery, using HRV as a physiological marker. Participants included 24 individuals who engaged in social drinking and 21 participants with AUD, all recruited from the Greater New Haven area. They were screened for psychiatric or medical conditions and other substance use disorders using DSM-5 criteria. Results showed significant main effects of stress condition and time across HRV metrics. While no significant three-way interaction among time period, condition, and drinking group was observed, there were significant condition × time period effects and group × condition effects. Participants with AUD exhibited lower high-frequency (HF) and low-frequency (LF) HRV during stress exposure compared to the recovery phase. They also showed a less dynamic LF/HF ratio during stress relative to social drinking controls, suggesting greater sympathetic dominance. In contrast, participants who engaged in social drinking displayed autonomic flexibility across time periods. Findings suggest that individuals with AUD experience blunted autonomic reactivity and reduced HRV recovery following stress, highlighting diminished physiological flexibility potentially indicating risk for long-term stress-related chronic diseases. The results underscore the importance of evaluating autonomic function in clinical care and recovery planning for individuals with AUD.

## 1. Introduction

Stress is far more than a fleeting emotional experience; it can mobilize powerful biological forces that, with chronic exposure, have the capacity to reshape health across multiple domains. Extensive research demonstrates that prolonged exposure to stress disrupts brain function, weakens immune defenses, and destabilizes key physiological systems (Yaribeygi et al., 2017). Over time, these disruptions erode protective mechanisms, increasing vulnerability to chronic illnesses such as cancer (Dhabhar, 2014), diabetes (Hackett & Steptoe, 2017), and cardiovascular disease (Steptoe & Kivimäki, 2012; Yusuf et al., 2004). Stress has also been linked to structural alterations in brain regions involved in memory, emotion and cognition (Ansell et al., 2012; Lupien et al., 2009; Sarahian et al., 2014), highlighting its role in shaping not only physical health but also cognitive and emotional processing and stress regulation capacities. The psychological consequences are equally profound, with adverse life events heightening risk for depression (Hammen, 2015) and substance use disorders (Sinha, 2008). Taken together, these findings underscore that stress is not a peripheral factor in health and disease, but a central pathway through which physical and mental health outcomes emerge. Studying stress and stress responses, therefore, is essential for understanding the biological mechanisms underlying many chronic conditions.

Given that stress has such widespread effects on brain, body, and behavior, heart rate variability (HRV) provides a particularly valuable window into its impact. HRV refers to the natural variation in the time interval between consecutive heartbeats—for example, even if the heart averages 60 beats per minute, the time between individual beats might vary slightly (e.g., 0.95 s, then 1.05 s, then 0.98 s). These moment-to-moment changes reflect how flexibly the autonomic nervous system is responding to environmental demands. By capturing the balance between sympathetic and parasympathetic activity, HRV not only reflects immediate autonomic responses to stress but also offers insight into an individual’s capacity for physiological flexibility and resilience (Sztajzel, 2004). HRV can be quantified using time-domain metrics (e.g., root mean square of successive differences (RMSSD) or frequency-domain metrics that break the signal into different frequency bands (Cygankiewicz & Zareba, 2013). Time-domain metrics include the RMSSD, which quantifies the short-term, beat-to-beat variability in heart rate, and standard deviation of normal to normal interval (SDNN), which captures overall HRV across a longer window of time. RMSSD reflects rapid parasympathetic modulation and is sensitive to immediate physiological changes such as deep breathing or acute stress, whereas SDNN captures the total autonomic variability and is interpreted as broader index of autonomic health. Frequency-domain metrics like low frequency (LF), high frequency (HF), and their ratio (LF/HF) divide the HRV signal into components based on how quickly the oscillations occur (Shaffer & Ginsberg, 2017). High frequency (~0.15–0.40 Hz) represents fast, breath-to-breath fluctuations in heart rate and is a direct marker of parasympathetic activity (Shaffer & Ginsberg, 2017), increasing during restorative states like sleep. Low frequency HRV (~0.04–0.15 Hz) reflects slower autonomic rhythms influenced by both the sympathetic and parasympathetic branches of the autonomic nervous system. LF is often used with HF to calculate the LF/HF ratio to provide an index of autonomic balance. Importantly, many studies show that HRV shifts in predictable ways under stress: RMSSD and HF typically decrease, while the LF/HF ratio increases (Kim et al., 2018; Laborde et al., 2017; Thayer et al., 2012). This pattern reflects sympathetic dominance—a state in which the sympathetic nervous system (“fight-or-flight”) becomes more active than the parasympathetic system (“rest-and-digest”)—indicating that the body is operating as if it needs to react, defend itself, or stay on high alert.

Decreased HRV, as measured by HF and RMSSD, has been associated with a diminished parasympathetic response and correlated with poorer health, whereas high HRV indicates an increased parasympathetic response (Das & O’Keefe, 2006; Thayer & Sternberg, 2006). Numerous studies have shown that HRV responds differently to stress versus non-stress conditions (Kim et al., 2018; Pereira et al., 2017). Specifically, stress may decrease RMSSD and HF markers of HRV and increase the LF/HF ratio, indicating sympathetic dominance (Hjortskov et al., 2004; Sloan et al., 1994). Difficulty returning to a homeostatic setpoint in HRV after experiencing a stressor might lend risk for the development of chronic diseases. Thus, HRV is a sensitive biomarker of autonomic regulation and a valuable tool for understanding how the body responds and adapts to stress.

Studies have employed various tasks to assess the impact of stress on HRV in broader populations. For example, cognitively demanding tasks result in increases in LF and the LF/HF ratio as well as decreases in HF in a healthy population (Held et al., 2021; Hjortskov et al., 2004). HRV is also affected by laboratory-based tasks that are designed to directly elicit an acute stress response. For example, the most popular acute stress provocation, the Trier Social Stress Test (TSST) (Kirschbaum et al., 1993), which includes a social evaluative stress component, demonstrates a reduction in HRV (Pereira et al., 2017; Sghir et al., 2012) and an increase in heart rate (Patock-Peckham et al., 2022). Another standardized and widely used stress task, the Cold Pressor Test (CPT), involves subjects immersing one of their hands in ice-cold water for 90 s to 3 min. The CPT has been commonly used to measure autonomic system functioning under stress (Lamotte et al., 2021), demonstrating decreased HRV and increase blood pressure (Macartney et al., 2021; Prakash et al., 2021). One attractive component of the classic CPT is that it does not include a social evaluative component, thereby bypassing a need for stress appraisal. Individuals undergoing the TSST, for instance, may respond differently based on a number of baseline characteristics that might decrease the salience of the social-evaluative threat (Sinha, 2024; Sinha et al., 2009; Stellern et al., 2023; Thomas et al., 2008). However, because of its brief nature, the standard CPT does not elicit an acute sustained stress response that makes the TSST a robust stressor. Therefore, there is room for improvement to broadly and robustly assess a multifaceted stress response salient to multiple populations.

Like stress, alcohol use can acutely impact HRV and autonomic regulation, which may have long-term implications for cardiovascular health (Romanowicz et al., 2011). Acute alcohol use in healthy individuals may decrease HRV and increase the LF/HF ratio, indicating sympathetic activation (Romanowicz et al., 2011; Spaak et al., 2010). Some evidence suggests a J-shaped curve relationship, where low to moderate alcohol use has protective effects, but higher doses may lead to poor health outcomes (Quintana et al., 2013). However more recent studies have refuted that there are benefits at any level of alcohol use, resulting in the World Health Organization taking the stance that there is no safe level of alcohol use (Anderson et al., 2023; Zhao et al., 2023). Few studies have examined the relationship between alcohol use disorder (AUD) and HRV, finding that individuals with AUD generally exhibit lower resting HF-HRV (Irwin et al., 2006, p. 200; Murata et al., 1994). This underscores the importance of examining how chronic alcohol use, especially in individuals with AUD, shape autonomic function, particularly in response to stress.

Previous studies have used various acute stress provocation tasks to explore the relationship between alcohol use, stress, and cardiovascular health. An acute laboratory social evaluative stressor increases alcohol craving, heart rate, and cortisol response (McCaul et al., 2018; Starcke et al., 2013). Individuals with alcoholic neuropathy have also exhibited both parasympathetic and sympathetic dysfunctions under acute stress (Chida et al., 1994, p. 199). Subjects with AUD have been noted to have increased heart rate under stress conditions (Hwang et al., 2021). Alcohol consumption has been directly associated with a greater blood pressure response to the CPT (Zhang et al., 2013). Despite these findings, few studies have examined autonomic function using the CPT in patients with AUD.

The Yale Pain Stress Test (YPST) (Fogelman et al., 2025) builds on the standard CPT to produce a robust stress response without a social evaluative component (see Table 1 for a comparison of commonly use stress tasks). This method is described in length in Fogelman et al. (2025) but we have described the YPST briefly here. The YPST employs a unique behavioral pain tolerance paradigm with three consecutive trials each that are 180 s in length where participants submerge their hand in ice-cold water for the pain-stress condition and immerse in warm water for the no pain-stress condition. In the current study, we sought to determine whether the YPST would result in dampened parasympathetic arousal (i.e., HF, RMSSD) and heightened sympathetic arousal (i.e., LF/HF ratio) during the cold water/pain-stress compared to the no pain-stress/warm water condition. In addition, we hypothesized that individuals with AUD would have dampened HRV arousal to the stress condition compared to individuals who socially drank.

## 2. Methods

### 2.1. Participants

The study included 24 individuals who drank alcohol socially (SD) and 21 individuals diagnosed with current AUD. Participants were recruited from the greater New Haven area and neighboring communities via flyers, social media ads, and online classified postings. All individuals in the study had to have a prior history of drinking alcohol. Individuals were classified as having AUD or a social drinker based on meeting criteria for AUD using the Structured Clinical Interview for DSM-5 (First et al., 2015). Individuals with AUD were enrolled regardless of treatment-seeking status; however, individuals were referred to appropriate treatment providers if they were treatment seeking. Exclusion criteria were the (1) presence of acute psychiatric symptoms or (2) any self-reported medical or psychiatric issues that required ongoing care. Additionally, subjects were excluded if they had current severe other substance use disorders (excluding cannabis) according to diagnosis, self-report, or urine toxicology screens. The study was approved by and carried out in compliance with the Institutional Review Board (IRB).

### 2.2. Procedures

**Figure 1:**
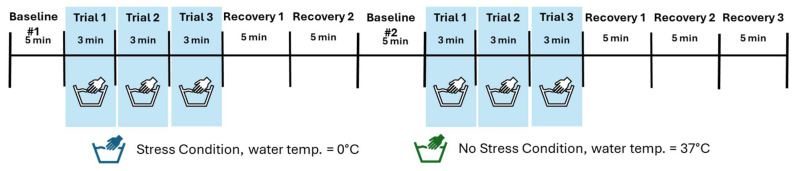
Schematic of the Yale Pain Stress Test (YPST) protocol used to assess autonomic response to stress. Participants completed three 3 min trials in two conditions: a Stress Condition involving cold water hand submersion and a No-Stress Condition involving room temperature water. Each trial was identical in timing and structure, allowing for within-subject comparisons of autonomic regulation across thermal stress and control conditions.

Potential participants were initially screened by phone to determine preliminary eligibility. Individuals were then invited to an in-person intake visit where informed consent was obtained. These appointments included self-report and interview assessments to assess eligibility further, gather demographic information, and provide baseline measures of alcohol use. Following the intake phase, subjects were scheduled for an afternoon laboratory visit to complete the YPST. Subjects were asked to abstain from alcohol use that day and to eat a light lunch at noon on the day of the experimental session and arrive for the session by 1:30 pm. Upon arrival at the Yale Stress Center, participants underwent urine drug screening to rule out recent drug use and verify their Timeline Follow Back results. Participants were instructed to attach the HRV recording device using two electrodes under the supervision of a research assistant. They were then informed about the procedure (see Figure 1 for procedures), which involved submerging their hand in warm/room-temperature water (37 °C, No Pain-Stress Condition) or cold water (0 °C, Pain-Stress Condition) for up to three consecutive trials, each being a maximum of 180 s each. The stress and no stress conditions were presented in a randomized, counterbalanced order on the same day. Ice chips were included in the water bath to maintain the 0 °C for the pain-stress condition and a temperature gauge assessed both buckets throughout. HRV was continuously monitored throughout the session. Participants were instructed as follows: “This is the warm-cold water test. I will ask you to submerge your hand up to your forearm in the water between 1–3 times. You will need to submerge your hand from your fingertips up through your wrist. We need you to keep it in as long as you can. We will record the length of time your hand is submerged. Let me know when you’re ready.” If asked about time remaining during the trial, the researcher informed the participant that they could not tell them but would let the participant know when to take it out. Participants kept their hand in the water for the entire 3 min trial period or if they could no longer tolerate the sensation. Informing participants that the length would vary from 1–3 times was to allow for an element of unpredictability. All participants were asked to submerge their hands in the water buckets for all three trials for a total of 9 min, thus resulting in task length comparable to the Trier Social Stress Task or the MAST (Kirschbaum et al., 1993; Smeets et al., 2012).

### 2.3. Measures

During intake appointments, demographic information, stress assessments, and general patterns of substance use in the last 30 days were collected. Drinking and substance use history were documented using the Timeline Follow Back (TLFB) method, a validated, calendar-based interview in which participants reconstruct their daily alcohol and drug use over a specified period using memory cues such as holidays, work schedules, or personal events. This method provides reliable estimates of quantity and frequency of use over time and is widely used in alcohol and addiction research (Sobell et al., 1996).

The standardized Structured Clinical Interview for DSM (SCID) was used to determine whether participants met the criteria for an exclusionary psychiatric condition or substance use disorder other than AUD (First et al., 2015).

The Alcohol Use Disorders Identification Test (AUDIT) (α = 0.88) was administered by a trained research assistant during intake procedures as an additional measure of alcohol use severity for both social drinkers and individuals with AUD (Saunders et al., 1993). The AUDIT is scored such that it provides cut-offs that indicate low-risk consumption (scores 0–7), hazardous or harmful alcohol consumption (score 8–14), and 15 or more indicating likely alcohol dependence (Babor et al., 2001).

**Figure 2:**
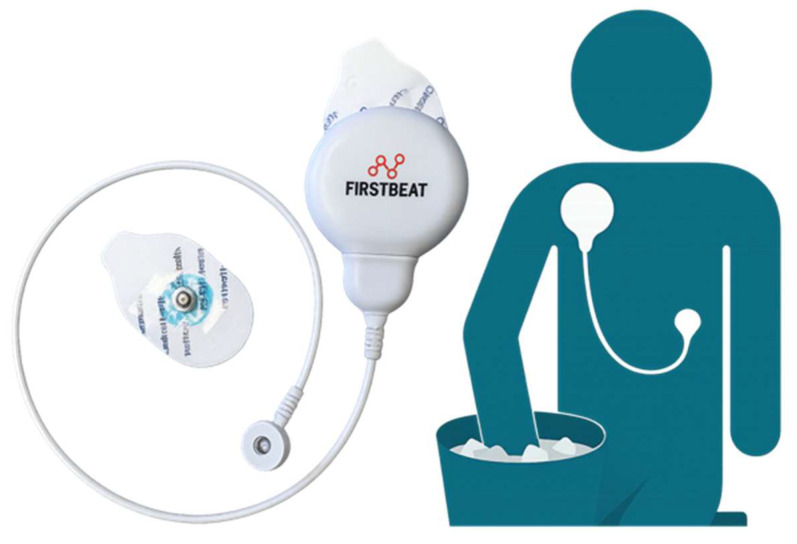
Firstbeat Bodyguard 2 device for capturing HRV and its placement on the body. The Firstbeat Bodyguard 2 is a medical-grade ECG device designed for high-precision HRV assessment. This device records beat-to-beat R-R intervals using two adhesive electrode patches placed on the chest. The image shows the device (**left**) and its corresponding placement on the body (**right**): one electrode is positioned below the right clavicle and the second on the lower left ribcage. A short, flexible cable connects the two electrodes.

HRV was recorded using a Firstbeat Bodyguard 2 Device (1000 Hz, Figure 2). Data were first pre-processed using the Firstbeat artifact correction and subsequently processed in the Kubios software (version 4.1.2). The baseline period was defined as the initial five minutes preceding the start of the Yale Pain Stress Test (YPST). Time intervals during the YPST were segmented into periods when the participants’ hands were in the water and periods when their hands were out of the water for each 180 s trial. Immediately following the three submersions, four five-minute recovery periods were identified. The following metrics were calculated: HF Power FFT (log-transformed), LF Power (log-transformed), LF/HF Ratio FFT, RMSSD, and SDNN. Log-transformed LF and HF metrics were used due to significant skewness in the raw HRV frequency power metrics.

### 2.4. Data Analytic Plan

Descriptive statistics were generated using R v3.6.0 with the dplyr and psych packages (Revelle, 2017; Wickham et al., 2025), and graphs were created using the ggplot2 and ggeffects packages (Lüdecke, 2018; Wickham, 2016). Statistical analyses were performed using R v3.6.0 with the lmer and emmeans packages (Bates et al., n.d.; Lenth, 2025). *T*-tests and chi-square tests were utilized to determine whether social drinkers and AUD patients differed on demographic and alcohol use characteristics. Changes in heart rate variability metrics during the YPST were modeled using linear mixed models with a specified random intercept, and Satterthwaite-approximated denominator degrees of freedom were employed. The main and interactive effects of the drinking group (AUD, SD), period within the trial, and condition were included as fixed effects. All models included relevant demographic variables (e.g., sex-assigned-at-birth, age in years) as covariates.

Prior work has established that the standard CPT paradigm has moderate to large effect sizes on physiological responses (Cohen’s d ≈ 0.3–0.5) with approximately 15–25 participants per group in a repeated measures design (al’Absi et al., 2021; Al’absi et al., 2012; Cooper & Haney, 2016; Kowalczyk et al., 2006), like what we used in the current study to test the proposed hypotheses. Past work that has examined the impact of acute stress on HRV has found a Hedge’s g of −0.530 (medium effect) on HRV metrics, including HF HRV and RMSSD (Brindle et al., 2014). Finally, a meta-analysis examining the effect of having AUD compared to control subjects on HRV metrics indicated a Hedge’s g = −0.43 (medium effect) (Cheng et al., 2019). Thus, we expected similar moderate to large effect sizes to detect condition and AUD status effects with a sample of 45 individuals in HRV response to stress with a power of 0.80 and a *p*-value set at 0.05, with expected adequate power to test the proposed hypotheses.

## 3. Results

### 3.1. Demographic Characteristics

Baseline drinking behavior and participants’ demographic and clinical characteristics are included in Table 2. Individuals with AUD were older, but no other demographic differences were significant. As expected, we found differences in alcohol involvement between groups: individuals with AUD had more years of regular alcohol use (3 or more drinking days per week; *p* < 0.001), more drinking days in the 30 days before starting the study (*p* < 0.001), a higher AUDIT score (*p* < 0.001), and a higher average number of drinks consumed in a drinking episode (*p* < 0.01) as compared to SD.

### 3.2. HRV Response to YPST

**Figure 3:**
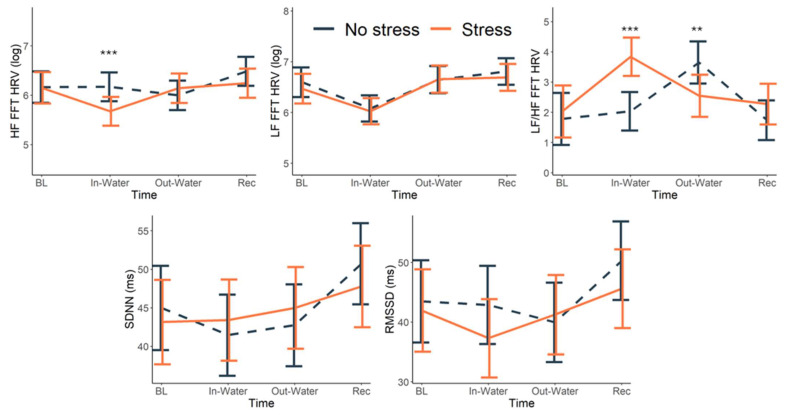
Yale Pain Stress Task Effects on HRV Metrics. Mean HRV metrics across time points (Baseline, In-Water, Out-of-Water, Recovery) during stress (orange, solid line) and no-stress conditions (dark blue, dashed line). Top panels show high-frequency (HF) and low-frequency (LF) power (log-transformed), LF/HF ratio (log-transformed), bottom panels show RMSSD (ms) and SDNN. Error bars represent ±1 SE. Significant condition effects were observed for HF HRV and LF/HF ratio, with lower HF and higher LF/HF during stress trials compared to no-stress (** *p* < 0.01, *** *p* < 0.001). RMSSD showed a trend toward reduced parasympathetic activity during stress. These results indicate differential autonomic responses to acute stress exposure.

We first examined frequency metrics of HRV. The two-way interaction of condition by trial was significant for log-transformed HF, *F*(3, 703.01) = 5.71, *p* < 0.001, and the ratio of LF/HF HRV, *F*(3, 703.00) = 5.27, *p* < 0.002 (Figure 3). Specifically, HF HRV was lower when the participants’ hands were submerged in the water during the stress condition as compared to the no-stress, estimated marginal means (EMM) = −0.039, *SE* = 0.0007, *t* = −5.60, *p* < 0.001. In contrast, LF/HF ratio was higher when their hands were submerged during the stress condition than in the non-stress condition, EMM = 1.39, *SE* = 0.313, *t* = 4.450, *p* < 0.001, indicating sympathetic dominance during stress. In addition, the LF/HF ratio was lower after removing their hand from the water during the stress condition as compared to the no stress condition, EMM = −1.13, SE = 0.383, *t* = −2.96, *p* = 0.003, indicating that recovery autonomic processes were more at play following the stress. When examining the time-domain metrics for HRV, RMSSD tended to be higher during the non-stress trials than the stress trials, although this effect was trending, *F*(3, 703.03) = 2.49, *p* = 0.059. SDNN and LF were not significantly different across the two conditions, *p* ≥ 0.103.

### 3.3. Alcohol Misuse Moderating Effects

**Figure 4:**
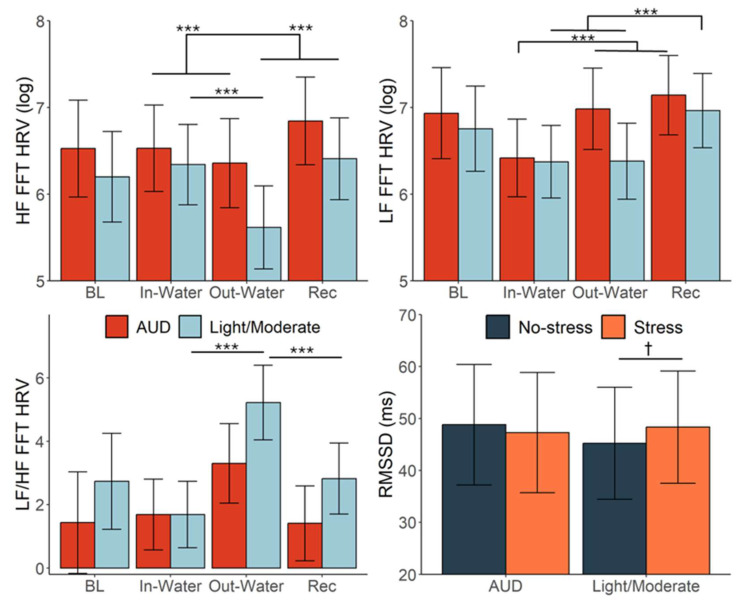
Individuals with AUD demonstrate a less dynamic HRV response compared to individuals who socially drink. Heart rate variability (HRV) metrics across experimental time points and conditions, separated by drinking group (participants with Alcohol Use Disorder [AUD], red; participants who drink at light/moderate levels, blue). Top panels show log-transformed high-frequency (HF) and low-frequency (LF) HRV, the bottom left panel shows LF/HF HRV ratio, and the bottom right panel shows RMSSD (ms) by stress condition (stress = orange; no-stress = dark blue). Significant time effects were observed for both groups, with participants with AUD showing blunted HF and LF HRV during task trials and a stronger recovery response compared to those who drank at light/moderate levels. Participants who drank at light/moderate levels exhibited greater sympathetic dominance (higher LF/HF ratio) during the out-of-water phase and reduced HRV overall during stress. † *p* < 0.10, *** *p* < 0.001. Error bars represent ±1 SE. These findings suggest altered autonomic recovery dynamics among participants with AUD.

No significant three-way interaction (trial × condition × drinking group) emerged for any HRV metric. However, a significant group × trial interaction was observed for the frequency-domain HRV metrics, including HF HRV, *F*(3, 703.01) = 3.96, *p* < 0.009; LF HRV, *F*(3, 703.00) = 3.54, *p* = 0.014; and the LF/HF ratio, *F*(3, 700.03) = 2.66, *p* = 0.048 (Figure 4). Among participants with Alcohol Use Disorder (AUD), HF HRV was significantly lower during the trial phase compared to recovery—both during hand submersion (EMM = −0.44, SE = 0.11, *p* < 0.001) and post-submersion (EMM = −0.30, SE = 0.12, *p* = 0.017). In contrast, participants who engaged in social drinking exhibited lower HF HRV when their hands were out of the water compared to when submerged (EMM = −0.40, SE = 0.11, *p* < 0.001) and when compared to recovery (EMM = −0.60, SE = 0.12, *p* < 0.001). Similarly, LF HRV among participants with AUD was lower during hand submersion relative to both the out-of-water trial (EMM = −0.60, SE = 0.13, *p* < 0.001) and recovery period (EMM = −0.70, SE = 0.12, *p* < 0.001). Participants who engaged in social drinking also showed reduced LF HRV during the task compared to recovery—submerged versus recovery: EMM = −0.56, SE = 0.11, *p* < 0.001; out of water versus recovery: EMM = −0.52, SE = 0.13, *p* < 0.001. For the LF/HF ratio, the LF/HF ratio for social drinkers was significantly higher during the out-of-water period compared to both the submerged trial (EMM = 2.09, SE = 0.50, *p* < 0.001) and recovery (EMM = 1.60, SE = 0.53, *p* = 0.003), but this response did not reach significance in participants with AUD (during the task compared to recovery—submerged: EMM = 0.93, SE = 0.51, *p* = 0.068; out of water: EMM = 1.09, SE = 0.56, *p* = 0.053). A significant condition × group interaction emerged for RMSSD, *F*(3, 703.03) = 5.14, *p* = 0.024 (Figure 3), driven by opposing tendencies in the simple effects. Social drinkers showed a non-significant trend toward higher RMSSD during stress versus non-stress (EMM = 2.63, SE = 1.56, *p* = 0.092), whereas individuals with AUD showed the reverse pattern, with lower RMSSD during stress (EMM = −2.02, SE = 1.47, *p* = 0.169).

## 4. Discussion

The present study highlights the utility of the YPST in eliciting robust autonomic responses to a standardized pain-stress challenge. Specifically, the YPST produced autonomic changes consistent with heightened sympathetic load, extending prior work showing that the YPST evokes a multi-level acute stress response (Fogelman et al., 2025). By intensifying the pain-stress provocation and embedding an element of uncontrollability—participants were not informed of how many immersions would be required—the YPST provides a meaningful expansion of the traditional cold pressor task, yielding responses comparable to other laboratory stress paradigms (Kirschbaum et al., 1993) without reliance on a social-evaluative component. Clear shifts in autonomic balance emerged across stress and recovery periods: parasympathetic activity, indexed by high-frequency HRV, was reduced during stress trials, while sympathetic dominance, reflected in the LF/HF ratio, was elevated during stress and returned toward baseline in recovery. Time-domain metrics revealed a similar pattern, with greater parasympathetic activity evident in non-stress conditions. These findings demonstrate that the YPST not only reliably induces sympathetic activation but also captures the transition into recovery, making it a valuable tool for probing individual differences in autonomic regulation under acute stress.

Importantly, these individual differences were evident when comparing participants with Alcohol Use Disorder (AUD) to those who drank socially. Individuals with AUD displayed attenuated variability in HRV across both provocation and recovery periods. This blunted pattern suggests a less flexible and adaptive autonomic response to acute stress, consistent with prior research documenting autonomic dysregulation in AUD populations (Gao et al., 2025; Ralevski et al., 2019). Whereas social drinkers demonstrated clear shifts in autonomic balance between sympathetic activation during stress and parasympathetic rebound during recovery, individuals with AUD showed diminished responsiveness, suggesting a physiologic stress response system that is dysregulated and constrained in its capacity to adapt. We did not find a three-way interaction of stress, drinking group, and recovery period, which constrains our ability to draw firm conclusions about differential time-dependent stress reactivity between the groups. Nevertheless, the significant two-way effects of group × condition (RMSSD) and group x trial (HF, LF, LF/HF) suggest differential autonomic regulation patterns between individuals with AUD and those who drink socially, with individuals with AUD showing blunted HRV reactivity and impaired recovery, suggesting reduced physiological flexibility in response to acute stressors.

A person’s ability to flexibly shift between sympathetic and parasympathetic activation is key to supporting adaptative responses to stressors (Berntson et al., 1993). In individuals with AUD, this reciprocity may be further disrupted. According to polyvagal theory, higher baseline vagal activity is associated with greater capacity for emotion regulation, whereas lower vagal activity is linked to reduced regulatory capacity (Porges, 2007). Altered autonomic responding is associated with increased involvement with addictive behaviors, such as smoking (Ashare et al., 2012) and alcohol (Hwang et al., 2021). Viewed in this context, the attenuated parasympathetic activity observed in the current study—alongside blunted LF/HF responsiveness—may reflect discoordination between the two branches of the autonomic nervous system or inappropriate parasympathetic activation during stress. Such reductions in parasympathetic dynamics have been associated with increased alcohol use during follow-up after abstinence (Eddie et al., 2023) and may contribute to difficulties regulating arousal in stressful situations, thereby increasing the likelihood of stress-related drinking and relapse. This lack of responsiveness mirrors our earlier observations of blunted HRV recovery during sleep in AUD (Wemm et al., 2023), suggesting that reduced autonomic flexibility may represent a physiological marker of relapse vulnerability.

The altered autonomic responses observed in individuals with AUD may reflect broader disruptions in stress-regulation systems. Chronic alcohol use has been linked to dysregulation of the hypothalamic–pituitary–adrenal axis, and structural and functional alterations in prefrontal–striatal circuits that normally support self-regulation (Blaine & Sinha, 2017; Sinha, 2024; Sinha et al., 2016). Reduced variability in HRV during acute stress may therefore signal a loss of adaptive flexibility within the autonomic nervous system, consistent with the concept of allostatic load (Thayer & Sternberg, 2006), where repeated or prolonged stress exposure leads to maladaptive recalibration of physiological systems. Whether this blunted responsiveness represents an existing vulnerability that predisposes individuals to alcohol misuse, or a consequence of neurotoxic effects of prolonged alcohol exposure, remains an important question for future research.

These findings have several potential clinical implications. The ability of the YPST to elicit reliable adaptive autonomic stress responses suggests that it could serve as a standardized laboratory probe for identifying individual differences in stress sensitivity. In AUD populations, HRV reactivity and recovery patterns may function as biomarkers for relapse vulnerability, offering opportunities for personalized risk stratification. Moreover, HRV can be readily monitored using wearable devices, as has been carried out previously (Eddie et al., 2023; Wemm et al., 2023), raising the possibility of extending this work into real-world settings to capture dynamic fluctuations in autonomic regulation during daily life. Connecting the laboratory-derived stress responding with real-world dynamics provides the opportunity to develop an individual’s autonomic regulation profile across settings. In clinical settings, such markers could help identify individuals who may benefit from interventions that directly target stress regulation, including interventions including mindfulness to address stress, breathing interventions, HRV biofeedback (Lehrer & Gevirtz, 2014), or pharmacotherapies with known effects on autonomic balance (Fox et al., 2012). HRV patterns observed during controlled laboratory stressors may also inform personalized relapse-prevention planning, as individuals showing impaired recovery under stress may require enhanced coping-skills training or closer monitoring during high-risk periods.

A notable strength of this study lies in the use of the YPST—a novel, physiologically validated stressor that differentiates conditions based on physical pain stress or not) but also includes a well-defined baseline and post-pain, stress recovery period. The alternating trials of water immersion and rest allow for precise within-subject comparisons and minimize expectancy effects. Additionally, HRV is a dynamic and rapidly changing signal; the repeated-trial design of the YPST provided a unique opportunity to capture real-time changes in autonomic function across stress exposure and recovery phases.

Future studies may build on these findings by incorporating larger and more diverse samples, as well as longitudinal designs assessing whether blunted HRV responsiveness improves with treatment or prospectively predicts relapse. Together, these directions may help to refine the use of HRV as both a mechanistic marker and a clinically actionable biomarker for stress-related risk and resilience in addiction.

Several limitations should be noted. First, the relatively small sample size limits generalizability. While we were adequately powered for medium-sized hypothesized effects, the smaller sample size might also have inhibited our ability to test higher-order effects. Thus, future research with larger, more diverse samples is needed to validate these findings. Second, due to the modest sample and low numbers of women, we were unable to assess sex-assigned-at-birth differences. The modest sample size also limited our ability to examine the effects of race-related discriminatory effects, such as microaggressions, or other systemic stressors on HRV responding to a non-social evaluative stressor. This line of research would be important to examine further. A final limitation is that the two groups differed in age. Although age was included as a covariate to mitigate potential confounding, this difference may still have influenced physiological responses to the YPST. Future studies should examine YPST effects in samples more closely matched on age to clarify whether age-related factors contribute to the observed group differences.

In summary, we provide evidence that the YPST can be used to elicit dynamic autonomic responses that can be used to investigate flexible cardiovascular autonomic dysfunction in AUD populations. This well-controlled experimental study demonstrates altered autonomic responses to acute stress in individuals with AUD, characterized by less dynamic parasympathetic activity and increased sympathetic arousal compared to individuals who socially drink. These findings underscore the importance of considering autonomic function in the clinical characterization of AUD and support further exploration of HRV as a potential biomarker for stress sensitivity and treatment response in this population.

## Figures and Tables

**Table 1:** Comparison of Laboratory Stress Paradigms: YPST, CPT, MAST, and TSST.

Feature of Task	YPST	CPT	MAST	TSST
Physical Stress/Pain	✓	✓	✓	
Social Evaluative Stress			✓	✓
Unpredictability	✓		✓	✓
Uncontrollability	Partial control		✓	✓
Task Duration/ Immersion Trials	10 min/ 3 trials (180 s each)	90 s/ 1 trial	10 min/ 5 trials (60–90 s each)	10–15 min/ 1 trial, 2 tasks, 5 min each
Behavioral Outcome	✓	✓	✓	

The Yale Pain Stress Test (YPST) is a repeated-measures pain-stress paradigm involving exposure to physical pain (e.g., ice cold water) across multiple 3 min (180 s) trials, allowing within-subject assessment of physiological and behavioral stress reactivity. It does not include a social-evaluative component. The Cold Pressor Test (CPT) similarly evokes physical pain-related stress through a brief hand immersion in ice-cold water, typically used as a single 90 s trial physiological stressor. The Maastricht Acute Stress Test (MAST) combines physical and social-evaluative stressors by pairing cold-water immersion with timed mental arithmetic under observation, thereby eliciting both pain and performance stress. In contrast, the Trier Social Stress Test (TSST) is a purely social-evaluative stress paradigm involving public speaking and mental arithmetic before an evaluative panel and is generally administered as a between-subjects manipulation rather than a repeated-measures task. These distinctions clarify how the YPST extends traditional paradigms by offering a repeated, within-subject design for quantifying physical stress reactivity without confounding social-evaluative effects.

**Table 2:** Participant summary table.

Characteristic	AUD *N* = 21 ^1^	Light/Moderate *N* = 24 ^1^
Sex assigned at birth		
Male	11 (52%)	10 (42%)
Female	10 (48%)	14 (58%)
Age in years *	35.71 (9.36)	30.13 (7.63)
Education	14.79 (2.67)	15.69 (2.14)
Ethnicity		
African American	7 (33%)	7 (29%)
Asian	1 (4.8%)	3 (13%)
Caucasian	11 (52%)	9 (38%)
Hispanic	2 (9.5%)	2 (8.3%)
Multirace	0 (0%)	2 (8.3%)
Native American	0 (0%)	1 (4.2%)
Drinking Days in Last 30 Days ***	12.48 (10.01)	2.87 (2.62)
Avg Drinks per Episode **	5.40 (5.56)	1.47 (0.77)
AUDIT ***	14.15 (6.50)	2.18 (1.62)
AUDIT Category		
Low risk	4 (19.0%)	23 (95.8%)
Hazardous use	10 (47.6%)	1 (4.2%)
Harmful use	1 (4.8%)	0 (0.0%)
Possible dependence	6 (28.6%)	0 (0.0%)
Avg Years Alcohol Use ***	13.93 (9.47)	3.17 (6.73)
Days Last Drink Prior to Lab Session	20.05 (71.28)	43.08 (166.61)

Note: * *p* < 0.05; ** *p* < 0.01; *** *p* < 0.001, ^1^ n (%); Mean (SD); Avg = Average, AUDIT = Alcohol Use Disorders Identification Test. Continuous variables are given as Mean (SD) Range. Categorical variables are given as *N* (%) per column. Student’s *t*-tests and Fisher’s exact tests were conducted on continuous and categorical variables, respectively, to test for sex differences.

## Data Availability

Data and code is available upon request from the authors.
